# Endoscopic and external dacryocystorhinostomy (DCR) - which is better?

**DOI:** 10.5935/1808-8694.20120025

**Published:** 2015-10-20

**Authors:** Peter John Wormald, Renato Roithmann

**Affiliations:** Professor and Chairman Department of Otolaryngology Head and Neck Surgery, University of Adelaide, Adelaide, Australia; Professor of Otorhinolaryngology at the School of Medicine of the Lutheran University - RS - Brazil; Associate Scientific Staff, Department of Otolaryngology. Mount Sinai Hospital, University of Toronto, Ontario, Canada

The traditional approach for patient with epiphora and a blocked naso-lacrimal duct was and external approach Dacryocystorhinostomy (DCR). The technique was popularized by Toti in the early 1900s and has changed little since then^1^. It required an external incision in the medial canthus of the eye for access to the lacrimal sac.

Success rates for this procedure have varied widely with report of success as low as the 60% and in the hands of specialized oculoplastic surgeons as high as 95%. In the early 1990's McDonogh & Meiring^2^ published the first accounts of endoscopic endonasal DCRs and this has gained increasing popularity as the endonasal anatomy and endoscopic surgical techniques have improved. The most important development was the understanding of the anatomy of the lacrimal sac in relation to the anatomy of the lateral nasal wall^3^. Early descriptions showed the lacrimal sac to be sited below the axilla of the middle turbinate while more recent research showed that the lacrimal sac is sited about 8 to 10 mm above the axilla.

This has had implications for the endoscopic techniques developed to access the lacrimal sac as well as for the results obtained with the endoscopic techniques. Early results with the endoscopic DCR were not as good as the best results obtained by external DCR and this was due to the poor understanding of the endoscopic anatomy of the lacrimal sac^4^. These initial attempts at endoscopic DCR only opened the lower half of the lacrimal sac and therefore did not fully the principles of the external DCR which emphasized the need for full exposure of the sac and therefore full marsupialization of the sac into the lateral nasal wall. Once the anatomy was fully understood endoscopic DCR techniques were adapted and full marsupialization of the lacrimal sac was achieved and the results of this technique has been showed to be as good if not better than those that can be achieved in the best hands with the external technique^5^, ^6^.

The added advantage of the endoscopic DCR technique is that it leave no external scar, does not disrupt the orbicularis oculi muscle which is the main driver of the lacrimal pump and should produce superior results in patients with functional nasolacrimal duct rather than anatomical nasolacrimal duct obstruction. A joint work of the otolaryngologist and the ophthalmologist might be advantageous for the best possible management of the patient with epiphora.

The ophthalmologist plays an important role in the pre-operative evaluation of the epiphora. The main indication for sugery is lacrimal duct obstruction. So, other causes must be excluded (blepharitis, punctual abnormalities, lid malposition,…). During surgery, the ophthalmologist might also help to correct concomitant canaliculus or punctal abnormalities and to canulate the lacrimal sac with a lacrimal probe.

The otolaryngologist might correct concomitant nasal and sinus abnormalities such as nasal septum deviation, nasal polyps and adenoids. The mucosal flaps, the lacrimal bone removal and the full exposure of the lacrimal sac are nicely performed under endoscopic assistance. Both the ophthalmologist and the otolaryngologist participate in the post operative care period. Endonasal endoscopic DCR with full sac exposure is now considered the gold standard for the management of nasolacrimal duct obstruction and is increasingly been practiced both by ophthalmologists and rhinologists^5^, ^6^.
